# The Origin of Intraspecific Variation of Virulence in an Eukaryotic Immune Suppressive Parasite

**DOI:** 10.1371/journal.ppat.1001206

**Published:** 2010-11-24

**Authors:** Dominique Colinet, Antonin Schmitz, Dominique Cazes, Jean-Luc Gatti, Marylène Poirié

**Affiliations:** 1 Institut National de la Recherche Agronomique, ESIM, INRA PACA, UMR 1301, Sophia Antipolis, France; 2 Centre National de la Recherche Scientifique, CNRS, UMR 6243, Sophia Antipolis, France; 3 Université Nice Sophia Antipolis, UFR Sciences, Sophia Antipolis, France; Stanford University, United States of America

## Abstract

Occurrence of intraspecific variation in parasite virulence, a prerequisite for coevolution of hosts and parasites, has largely been reported. However, surprisingly little is known of the molecular bases of this variation in eukaryotic parasites, with the exception of the antigenic variation used by immune-evading parasites of mammals. The present work aims to address this question in immune suppressive eukaryotic parasites. In *Leptopilina boulardi*, a parasitic wasp of *Drosophila melanogaster*, well-defined virulent and avirulent strains have been characterized. The success of virulent females is due to a major immune suppressive factor, LbGAP, a RacGAP protein present in the venom and injected into the host at oviposition. Here, we show that an homologous protein, named LbGAPy, is present in the venom of the avirulent strain. We then question whether the difference in virulence between strains originates from qualitative or quantitative differences in LbGAP and LbGAPy proteins. Results show that the recombinant LbGAPy protein has an *in vitro* GAP activity equivalent to that of recombinant LbGAP and similarly targets Drosophila Rac1 and Rac2 GTPases. In contrast, a much higher level of both mRNA and protein is found in venom-producing tissues of virulent parasitoids. The F1 offspring between virulent and avirulent strains show an intermediate level of LbGAP in their venom but a full success of parasitism. Interestingly, they express almost exclusively the virulent LbGAP allele in venom-producing tissues. Altogether, our results demonstrate that the major virulence factor in the wasp *L. boulardi* differs only quantitatively between virulent and avirulent strains, and suggest the existence of a threshold effect of this molecule on parasitoid virulence. We propose that regulation of gene expression might be a major mechanism at the origin of intraspecific variation of virulence in immune suppressive eukaryotic parasites. Understanding this variation would improve our knowledge of the mechanisms of transcriptional evolution currently under active investigation.

## Author Summary

Evolutionary theories that discuss evolution of virulence in parasite species rely on the assumption that there is additive genetic variation for virulence traits, and that some alleles can then be readily selected, for instance following changes in host resistance genotypes. However, the molecular bases of this variation remain to be deciphered to better estimate the potential for evolution of virulence. This approach has been fruitful to understand evolution of insect resistance to insecticides, with point mutations, gene amplification and changes in expression level as possible sources of genetic variation. Parasitoids, auxiliaries used for biological control of insect pests, provide excellent models to study the coevolutionary processes that may drive changes in parasite host range. We describe here for the first time a mechanism at the origin of the intraspecific variation of virulence in a parasitoid wasp, a model for immune suppressive eukaryotic parasites, through regulation of the transcription of a major virulence factor. This study represents a new step in understanding both the evolutionary origin of virulence factors and their intraspecific variation, which may help optimize biological control success in the field.

## Introduction

Models of host-parasite coevolution all assume occurrence of genetic variation for host resistance and parasite virulence, such as well-described for plant-pathogen interactions [1] and in a few host-parasite models [2] [3]. Advances in understanding coevolutionary interactions thus require unraveling the molecular bases of host resistance and parasite virulence, and then acquiring data on how polymorphism in genes controlling these traits affect parasitism success in the field [4]. This will enable direct observation, rather than inference, of the host-parasite coevolutionary dynamics.

One largely unaddressed question in eukaryotic parasites is the basis of intraspecific variation of virulence. Regarding immune aspects, the only described mechanism is, to our knowledge, the antigenic variation used by fungal and protozoan parasites to hide themselves from the mammalian immune system [5]–[10]. Whether changes associated with virulence variation in immune suppressive parasites are qualitative and/or quantitative and what is their nature, still remain to be assessed.

The biological models of interacting species that satisfy all the requirements to study coevolutionary processes, from molecular tools to population polymorphisms, are scarce and *Drosophila*-parasitoid wasps interactions are undoubtedly among the best of them [11]. Variation in both *Drosophila* resistance and parasitoid virulence is observed in the field [12]–[14], and some major genes involved in these traits have been characterized [15]–[18]. Based on these data, we address here the question of the origin of intraspecific variation of virulence of *Drosophila* parasitoids.

Endoparasitoid wasps develop inside the body of their arthropod host, which will die as a result of the interaction [19],[20]. To face the immune defense of the host, they have evolved original strategies ranging from displaying surface features that prevent their recognition to altering components of the host immune system using venom proteins, virus-like particles or wasp-specific viruses, polydnaviruses [21],[22]. However, occurrence of virulence variation together with molecular identification and characterization of the factors involved in immune suppression has only been reported in the wasp *Leptopilina boulardi* [15],[16],[23],[24].

*Drosophila melanogaster* immune response to a parasitoid consists in the formation of a multicellular melanized capsule around the wasp egg that results in the death of the parasitoid [25]. Plasmatocytes first attach to the parasitoid egg and spread around it, then lamellocytes adhere to them to form multiple cell layers [26],[27].

Two types of *L. boulardi* wasps have been described based on their virulence properties against Drosophila hosts. The ISm isofemale line, highly virulent against *D. melanogaster* (and so-called “virulent” line) is representative of Mediterranean *L. boulardi* wasps both in its virulence properties and venom protein profile [23],[28]. It produces in its venom a RacGAP domain-containing protein, named LbGAP, whose injection in *D. melanogaster* larvae mimics the egg protection provided by parasitism [23],[24],[29]. LbGAP has a RacGAP activity and induces changes in the morphology of *D. melanogaster* lamellocytes [23],[24]. It specifically interacts with and inactivates two Drosophila Rho GTPases, Rac1 and Rac2 [15], both required for successful encapsulation of Leptopilina eggs [27],[30]. Interestingly, ISm wasps are not virulent against the host species *D. yakuba*, and, accordingly, host lamellocytes remain unchanged in presence of ISm venom [31].

By contrast, the success of the ISy isofemale line of *L. boulardi* depends on the host phenotype and it was then called “avirulent” [12],[13]. This line originates from Congo, where variation of parasitism success toward *D. melanogaster* was observed, due to occurrence of a polymorphism in the *L. boulardi* virulence phenotype [13],[28]. This polymorphism is likely due to the fact that several *Drosophila* host species are available in tropical Africa that can successfully be parasitized, and that virulence toward *D. melanogaster* may be costly for the parasitoid [13],[28]. Using the avirulent ISy line, resistant and susceptible *D. melanogaster* reference strains were obtained from a sympatric Congolese *D. melanogaster* population [32]. However, resistance to ISy parasitoids is not restricted to Congo but is present at high frequencies in tropical as well as Mediterranean host populations [13].

ISy parasitoids are able to suppress the immune defenses of susceptible larvae of *D. melanogaster* as well as of other host species such as *D. yakuba* [12],[16]. However, their parasitism success is not associated with an alteration of the host lamellocyte shape [24],[31]. Accordingly, at the protein level, no major band of the size of LbGAP was observed in electrophoretic analysis of ISy venom producing tissues [23]. Taken together, all these data point to LbGAP as a major virulence factor in Mediterranean *L. boulardi* parasitoids, involved in variation of parasitism success against *D. melanogaster*. In ISy tropical parasitoids, the venom contains a serpin that alters melanisation in the hemolymph of *D. yakuba* larvae [16], and might be responsible for the delayed encapsulation induced by injection of total venom [31]. The way ISy females counteract the immune defenses of susceptible *D. melanogaster* flies is yet totally unknown.

In this study, we first confirm that LbGAP is required for virulence against resistant *D. melanogaster* hosts. We demonstrate that both the virulent and avirulent parasitoid lines produce a RacGAP protein in their venom, and we report the sequence of the *RacGAP* gene homologous to *LbGAP* from the avirulent line (*LbGAPy*). We then compare the activity of LbGAP and LbGAPy proteins, their level of interaction with their Rac targets and their localization in the lamellocytes of parasitized hosts. Finally, we present quantitative data on LbGAP and LbGAPy at the mRNA and protein levels on virulent, avirulent, and F1 parasitoids. Results show that differences between virulent and avirulent parasitoids regarding the RacGAP toxin are only quantitative and very likely due to variation in *cis*-regulation of gene expression.

## Results

### LbGAP is necessary for *L. boulardi* virulence

**Figure 1:**
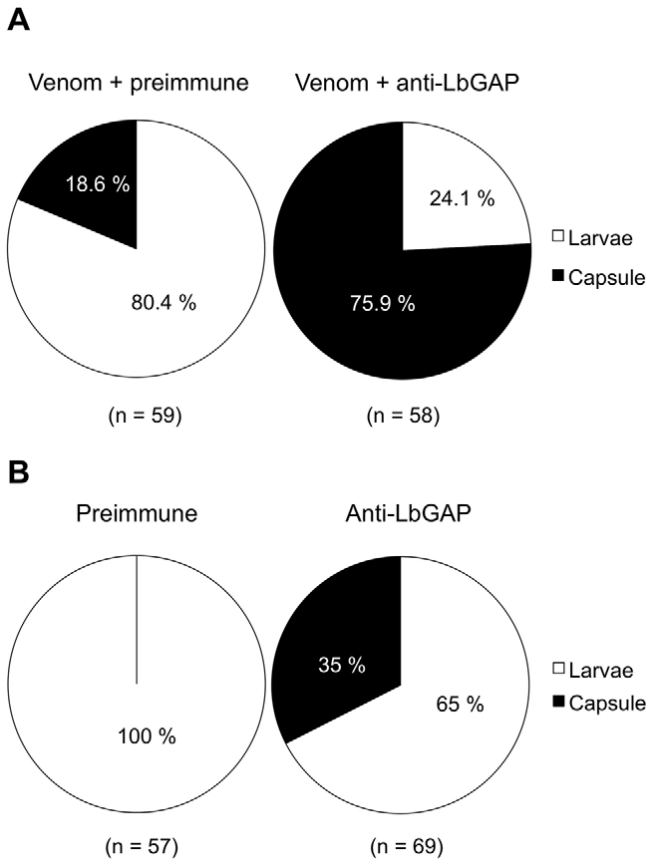
The RacGAP protein is necessary for virulence of ISm females. (**A**) *D. melanogaster* resistant L2 larvae were injected with ISm venom incubated either with the pre-immune serum as a control or a specific polyclonal antibody against LbGAP, then parasitized with the ISy avirulent line. The encapsulation rate was recorded after 48 h. In brackets are the numbers of injected larvae. (**B**) *D. melanogaster* resistant L2 larvae were injected with the pre-immune serum as a control or the specific antibody against LbGAP, and then parasitized with the ISm virulent line. The encapsulation rate was recorded after 48 h. In brackets are the numbers of injected larvae.

Previous work had shown that LbGAP is sufficient for successful parasitism of resistant *D. melanogaster* hosts by virulent parasitoid females. In order to determine whether this factor is also necessary for virulence, we performed experiments of injection of ISm venom in resistant *D. melanogaster* larvae, which is known to protect avirulent ISy eggs from encapsulation [24],[29]. In the present work, ISm venom was incubated before injection either with a specific polyclonal antibody against LbGAP or with the preimmune serum as a control, and larvae were then submitted to parasitism by ISy females. Venom incubated with the preimmune serum conferred active protection to avirulent eggs, with only 18.6% of encapsulation (Figure 1A). By contrast, incubation of ISm venom with the antibody against LbGAP led to 75.9% of avirulent eggs being encapsulated (Chi2 = 38.43 ; ddl = 1; p<0.001). A second experiment was performed in which the LbGAP antibody or the preimmune serum were injected alone into resistant host larvae that were subsequently parasitized by virulent ISm parasitoids. The encapsulation rates were 0% with the preimmune serum and 34.8% following injection of the LbGAP antibody, respectively (Chi2 = 24.49 ; ddl = 1 ; p<0.001; Figure 1B). These results demonstrate that LbGAP is a venom protein needed for *L. boulardi* virulence against resistant *D. melanogaster* flies.

### A *LbGAP* homolog in the ISy *L. boulardi* line

**Figure 2:**
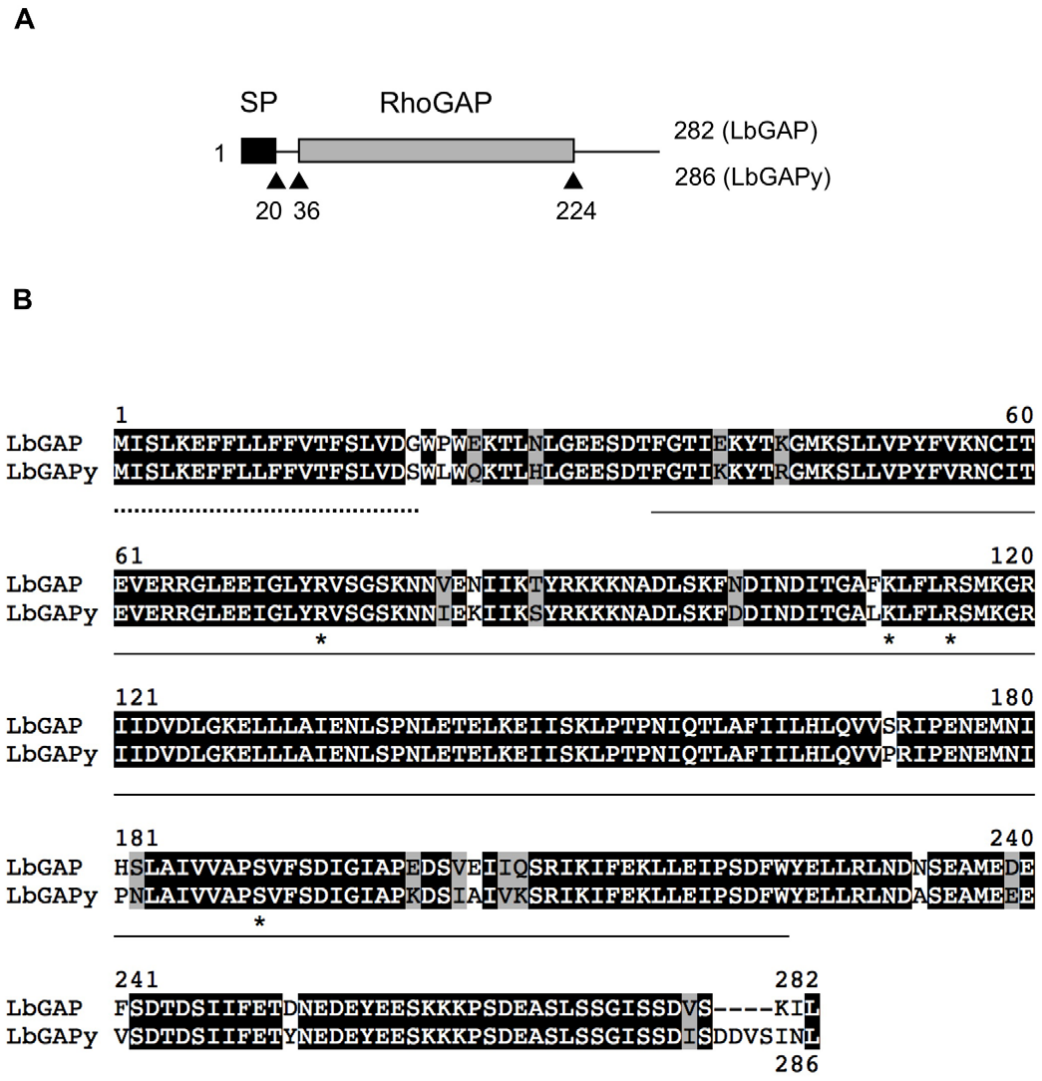
Sequence analysis of LbGAPy, the LbGAP homolog from the ISy *L. boulardi* line. (**A**) Schematic representation of LbGAP and LbGAPy amino acid sequences. The signal peptide (SP) and the RhoGAP domain are shown as black and gray rectangles, respectively. (**B**) Sequence alignment of LbGAP and LbGAPy amino acid sequences. Residues identical or similar are highlighted in black and grey, respectively. The signal peptide is indicated by a dotted line. The RhoGAP domain is underlined. Stars identify LbGAP residues involved in the interaction with Rac GTPases.

Whether an homolog of *LbGAP* is expressed in ISy parasitoids was questioned by performing PCR experiments on cDNAs from ISy venom-producing tissues, using primers designed from the sequence of *LbGAP* [23]. A 914 bp amplicon was obtained whose sequence contains a 861 bp ORF (GenBank accession number GU300066) encoding a predicted protein of 286 amino acids that was named LbGAPy (Figure 2A). Like LbGAP, this protein starts with a N-terminal signal peptide of 20 amino acids allowing its extracellular export, and it contains a RhoGAP domain. The nucleotide sequences of *LbGAP* and *LbGAPy* are 95.2% identical, while LbGAP and LbGAPy proteins share 89.5% identity and 94.8% similarity (Figure 2B). LbGAPy contains the four amino acid residues Arg74, Lys111, Arg115 and Ser190, described to be involved in LbGAP interaction with Rac GTPases [15]. Arg74 is conserved in all GAP proteins and forms an arginine-finger that stabilizes the GTPase invariant glutamine residue 61 or 63 to facilitate the catalysis of GTP to GDP [33]. The main difference between LbGAPy and LbGAP sequences is located at the C-terminal end of the protein outside the RhoGAP domain (Figure 2B).

### RacGAP activity of LbGAPy

**Figure 3:**
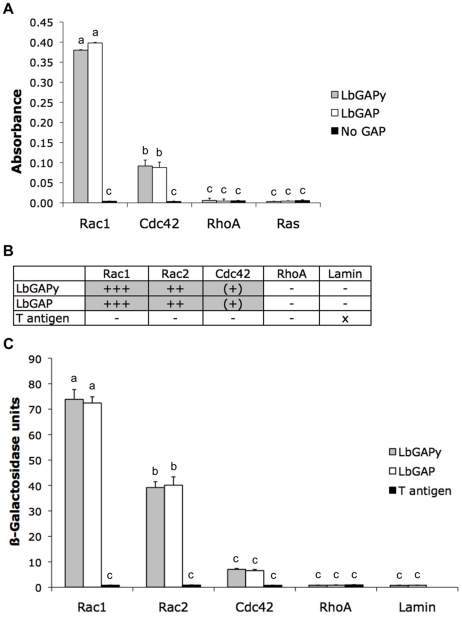
LbGAPy displays a GAP activity *in vitro* and interacts with *D. melanogaster* Rac1 and Rac2. (**A**) Absorbance at 650 nm, which is correlated with the amount of Pi released from GTP-bound human Rac1, Cdc42, RhoA or Ras, was measured in the presence of LbGAPy or LbGAP or in the absence of any GAP. Bars: GAP activity in the presence of LbGAPy (grey bars), in the presence of LbGAP (open bars), in the absence of GAP (black bars). For each value, error bars represent the standard error of three measurements. (**B**) Results based on growth on selective medium lacking histidine and qualitative ß-galactosidase overlay assays. x : non-tested; − : no interaction; (+) : very weak interaction; ++ : mean interaction, ++++ : strong interaction. (**C**) Interactions with Rac1G12V, Rac2G12V, Cdc42G12V, RhoAG14V and Lamin (negative control) assayed by measuring beta-galactosidase activity in total protein extracts. Grey bars : beta-galactosidase activity in the presence of LbGAPy. Open bars : beta-galactosidase activity in the presence of LbGAP. Black bars : beta-galactosidase activity in the presence of T antigen (negative control). For each value, error bars represent the standard error of three measurements.

LbGAP was previously shown to display a GAP activity with a strong preference for Rac-GTPases [15]. In order to determine if LbGAPy may have a similar GAP function in host cells, we carried out *in vitro* GAP assays using the LbGAPy protein produced in *E. coli*. Experiments were performed with human RhoA, Rac1 and Cdc42 Rho-GTPases. Human Ras, belonging to the Ras-GTPase family, was included as a negative control while LbGAP as well as the GAP domain from human p50 RhoGAP (which stimulates GTPase activities of RhoA, Rac1 and Cdc42 *in vitro*), were used as positive controls. Other negative controls consisted in the omission of either small G-protein or GAP protein. Similarly to LbGAP, LbGAPy significantly increased the GTPase activity of human Rac1 and Cdc42 but not of RhoA and Ras (F = 109.2; df = 11; p<0.001; Figure 3A). As for LbGAP, the GAP activity towards Rac1 was four times higher than towards Cdc42, suggesting that Rac-GTPases are the preferred substrates of LbGAPy as well.

### Physical interaction of LbGAPy with Rac1 and Rac2 GTPases

As LbGAP is known to specifically interact with *Drosophila* Rac1 and Rac2 [15], we questioned whether LbGAPy similarly targets these Rac-GTPases by performing yeast two-hybrid analyses. In order to stabilize interactions, we used the G12V mutated forms of *Drosophila* Rac1, Rac2 and Cdc42 GTPases and the G14V mutated form of *Drosophila* RhoA. Each of these mutants is deficient in GTPase activity and therefore constitutively blocked in the GTP-bound active conformation. Fusions of the GAL4 activation domain with LbGAP were expressed in yeast together with fusions of the LexA-DNA binding domain either with Rac1G12V, Rac2G12V, Cdc42G12V or RhoAG14V. Direct *in vivo* interaction of LbGAPy with small GTPases was measured as the ability of transformed yeast to activate the transcription of HIS3 and lacZ reporter genes, both under the control of the LexA-binding sequences. Yeast growth on a selective medium lacking histidine revealed that, similarly to LbGAP, LbGAPy interacts with Rac1G12V and Rac2G12V but only weakly with Cdc42G12V, and has no interaction with RhoAG14V (Figure 3B).

The strength and specificity of the interaction between LbGAPy and Rac GTPases was then estimated by titration of ß-galactosidase activity (Figure 3C). Substantial activity was seen using coexpression of GAL4AD-LbGAPy and either LexABD-Rac1G12V or LexABD-Rac2G12V but not in combination with non-specific sequences. The beta-galactosidase activity resulting from the interaction between LbGAPy and Rac GTPases was similar to that obtained using LbGAP and the same Rac GTPases (F = 92.2; df = 13; p<0.001; Figure 3C), thus demonstrating that this interaction is as strong and specific as the one demonstrated with LbGAP.

### Differences in LbGAP and LbGAPy amounts in host target cells

**Figure 4:**
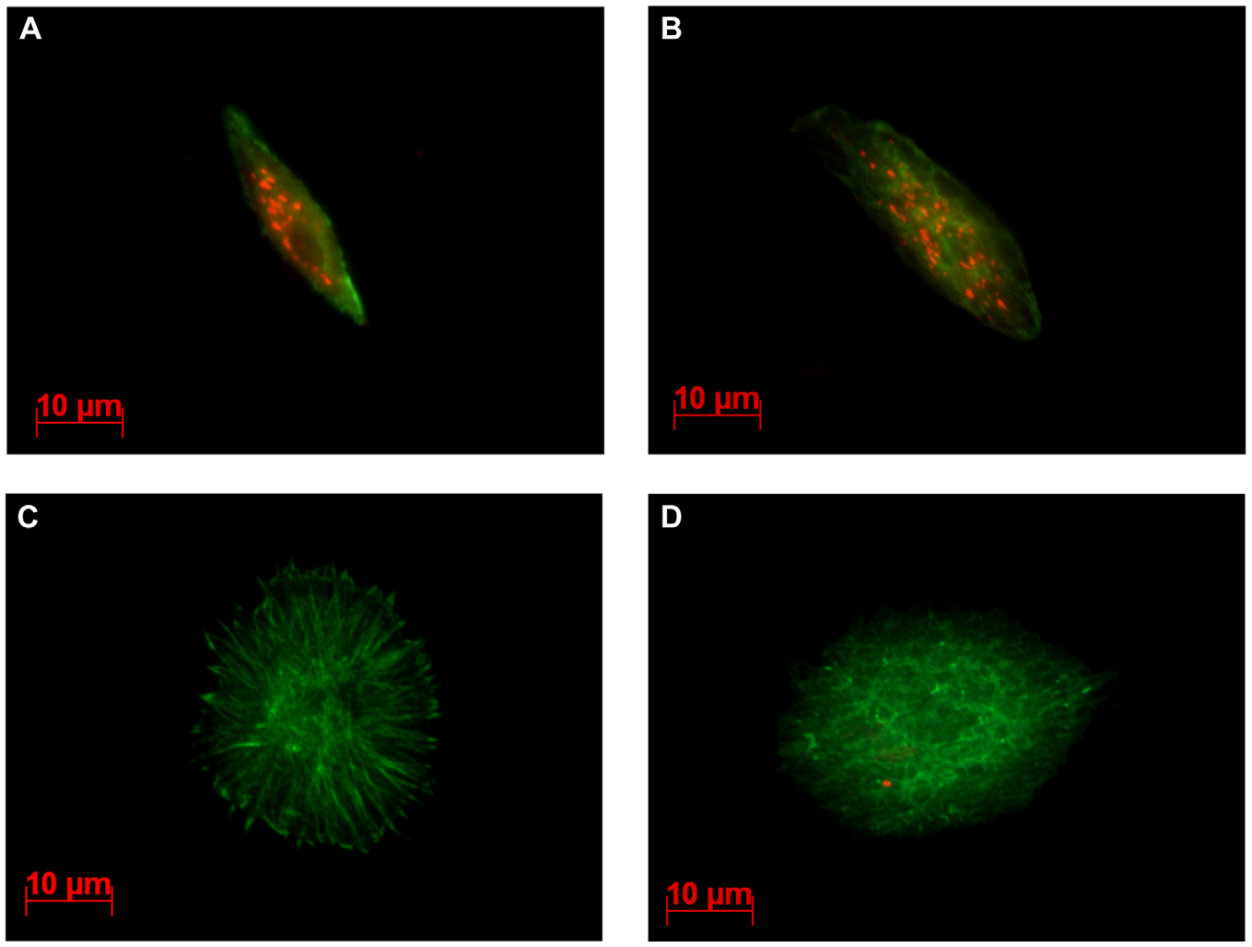
LbGAP and LbGAPy are detected in lamellocytes of parasitized *D. melanogaster* larvae. Immunocytochemical detection of LbGAP was performed on hemolymph collected from third-instar Drosophila larvae, 15hrs following parasitism. The LbGAP toxin is visualized as red spots while actin is detected by green labeled phalloidin. In larvae parasitized with ISm (**A** and **B**), most lamellocytes contain numerous LbGAP spots in their cytoplasm and their morphology is modified. In contrast, lamellocytes from larvae parasitized with ISy parasitoids are generally unchanged and without LbGAPy spots (**C**), a few of them containing a small number of LbGAPy spots (**D**).

We previously showed that LbGAP enters plasmatocytes and lamellocytes in ISm-parasitized *D. melanogaster* larvae and that morphological changes in lamellocytes are correlated with the intracellular quantity of LbGAP [15]. Using the polyclonal antibody raised against LbGAP that equally recognizes LbGAPy (see below), immunolocalization experiments were performed on hemolymph from *D. melanogaster* larvae 48 hours following parasitization by either ISm or ISy females. As expected, the majority of lamellocytes from ISm-parasitized larvae had a modified morphology and contained LbGAP (red intracytoplasmic fluorescent dots; Figure 4A and 4B). In contrast, lamellocytes from ISy-parasitized larvae remained largely unmodified and very few contained LbGAPy dots (Figure 4C and 4D). Among these, the number of dots was usually less than five whereas many lamellocytes from ISm-parasitized larvae contained more than 30 dots.

### Different levels of *LbGAP* and *LbGAP*y expression in venom-producing tissues and female residual bodies

**Figure 5:**
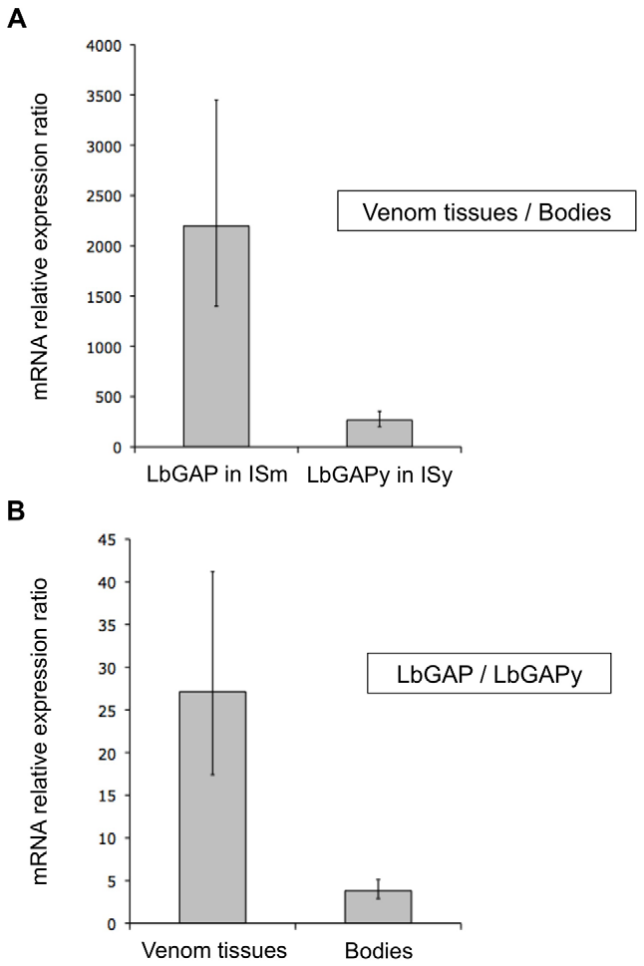
*LbGAP* expression in ISm females is higher than *LbGAP*y expression in ISy females. (**A**) Relative expression of *LbGAP* (ISm line) and *LbGAP*y (ISy line) in venom-producing tissues compared to the rest of the body without venom-producing tissues. (**B**) Ratio of relative expression of *LbGAP* (ISm line) compared to *LbGAP*y (ISy line) in venom-producing tissues and in the rest of the body without venom-producing tissues. For each value, error bars represent the standard error of three measurements.

qRT-PCR experiments were performed on ISm and ISy female samples of the same age to quantify differences in expression levels of *LbGAP* and *LbGAP*y genes between venom-producing tissues and the rest of the bodies, and to compare expression levels of *LbGAP* and *LbGAP*y in their respective parasitoid line. A 2200-fold higher expression was observed for *LbGAP* in ISm venom-producing tissues as compared to the rest of the female body (Figure 5A; Student t-test : t = −19.1, df = 12, p<0.001). Expression of *LbGAP*y was also higher in ISy venom producing tissues than in the rest of the body, but only with a 270-fold higher expression level (Figure 5A; Student t-test: t = −16.6, df = 10, p<0.001). When comparing parasitoid strains, *LbGAP*y was approximately 30 times less expressed in ISy venom-producing tissues than *LbGAP* in ISm venom-producing tissues (Figure 5B; Student t-test: t = −9.4, df = 15, p<0.001).

### No significant difference in *LbGAP* and *LbGAP*y gene copy number

To determine whether the difference in the number of *LbGAP* and *LbGAP*y transcripts results from a difference in the number of gene copy, qPCR experiments were performed on genomic DNA from the venom-producing tissues and from the rest of the female bodies of ISm and ISy parasitoids, respectively. No significant differences were found between the copy number of the *LbGAP* gene in ISm venom producing tissues and ISm residual female bodies or of the *LbGAP*y gene in ISy venom producing tissues and ISy residual bodies (df = 3, F = 1.4651, p = 0.2953).

### Differences in LbGAP and LbGAPy protein amounts in venom producing tissues

**Figure 6:**
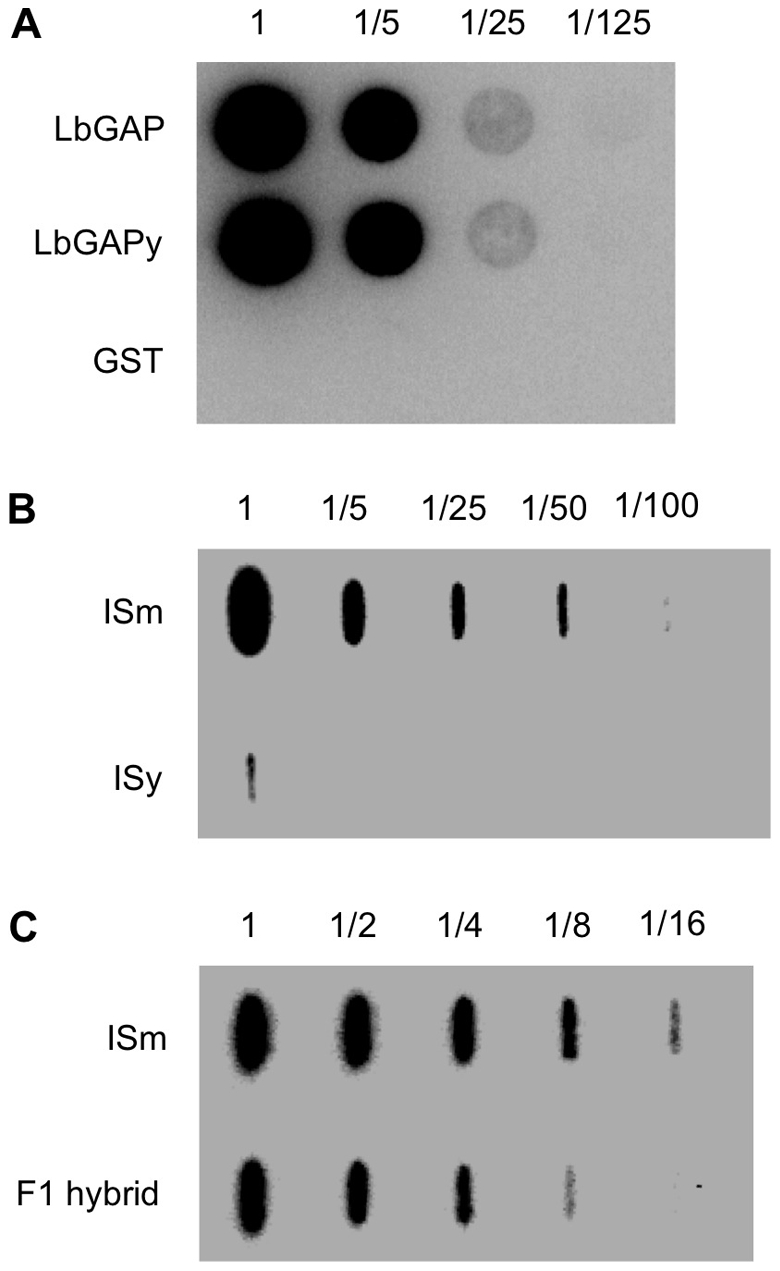
LbGAP amount in ISm females is higher than LbGAPy amount in ISy females. (**A**) Dot blot experiments on serial dilutions of the recombinant proteins GST-LbGAP and GST-LbGAPy using a LbGAP-specific polyclonal antibody. GST-tag alone was used as a control. (**B**) Slot blot on serial dilution of protein extract starting from twenty ISm and twenty ISy venom apparatus. (**C**) Slot blot on serial dilution of protein extract starting from five ISm and five F1 hybrid venom apparatus.

In previous Western blot experiments using a specific polyclonal antibody against the recombinant LbGAP protein, no signal was observed in ISy venom-producing tissues, possibly because the technique employed was not sensitive enough or because the antibody does not recognize LbGAPy [23]. These hypotheses were tested by producing both LbGAP and LbGAPy as GST-fusion proteins in *Escherichia coli* and using them to perform dot blot experiments on serial dilutions starting from the same quantity of these proteins. Our results show that the antibody recognizes specifically the LbGAP and LbGAPy recombinant proteins with the same efficiency (Figure 6A).

We then performed dot blot experiments with serial dilutions of total protein extracts from 20 ISm and 20 ISy venom-producing tissues (which represent the same amount of protein), using the anti-LbGAP polyclonal antibody. LbGAP was easily detected in ISm sample dilutions while LbGAPy could only be detected in the undiluted ISy extracts. The quantity of LbGAPy was then estimated to be 60 times less than that of LbGAP in ISm venom tissues (Figure 6B).

### A threshold effect of LbGAP quantity on virulence?

In order to further investigate the role of LbGAP quantity in ISm virulence, we performed crossing experiments between ISm females and ISy males and we assessed the virulence level of the F1 offspring, as well as the LbGAP/LbGAPy quantity in F1 venom-producing tissues, using dot-blot experiments. The amount of the LbGAP/LbGAPy proteins in F1 venom-producing tissues was approximately half the amount of LbGAP in ISm (Figure 6C). In parasitism experiments with the resistant strain of *D. melanogaster* in which ISy parasitoids are highly encapsulated (virulence level 5.7%), virulence of F1 hybrids (virulence level 98%) did not significantly differ from virulence of ISm parasitoids (virulence level 100% ; p<0.001).

### Variation of expression of *LbGAP* and *LbGAPy* is likely under the control of *cis*-acting elements

**Figure 7:**
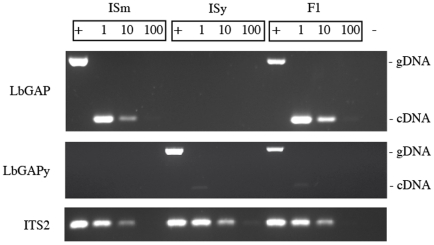
Variation of expression of *LbGAP* and *LbGAPy*. PCR experiments were performed on genomic DNA (+) to assess the specificity of the *LbGAP*- and *LbGAPy*-specific primers, and on serial dilutions of cDNA templates (1, 1/10, 1/100) to determine whether the variation of expression of *LbGAP* and *LbGAPy* is under the control of *cis*- or *trans*-acting elements. The *ITS2* ribosomal sequence was used as control to assess the quantity of the DNA and RNA samples. (−) Negative control.

To determine whether the variation of expression of *LbGAP* and *LbGAPy* is under the control of *cis*- or *trans*-acting elements, we performed PCR experiments on cDNA obtained from venom-producing tissues of ISm, ISy and F1 females. Two sets of primers were designed that respectively amplify *LbGAP* and *LbGAPy* and their specificity was tested using genomic DNA extracted from total bodies: a 670 bp and a 684 bp PCR product were amplified from ISm and ISy females, respectively, while both fragments were amplified from F1 individuals. Using ISm and F1 cDNAs and the *LbGAP*-specific primers, an intense band corresponding to a 252 bp amplicon was obtained. By contrast, only a faint band of 265 bp was observed using the *LbGAPy*-specific primers and cDNA from ISy or F1 individuals (Figure 7). Control of genomic DNA and cDNA quantities were performed using the internal transcribed spacer 2 (*ITS2*) gene.

Overall, these results show that F1 individuals overexpress the *LbGAP* and not the *LbGAPy* allele in venom tissues. This strongly suggests that variation of expression of *LbGAP* and *LbGAPy* is under the control of *cis*- rather than *trans*-acting elements.

## Discussion

Intraspecific variation in virulence occurs in several eukaryotic parasite species [31],[34]–[36], but it has only been explained in some mammalian parasites such as *Toxoplasma gondii*, *Trypanosoma brucei* or *Plasmodium falciparum*. In these species, antigenic variation occurs, mediated by the differential expression of surface molecules [5]–[10]. In parasitoid wasps, intraspecific variation in virulence has been reported in three species, *Asobara tabida*, *Cotesia sesamiae* and *L. boulardi*. In *A. tabida*, it is associated with a difference in immunoevasion capacities [35] in relation with the degree of embedment of the parasitoid egg in host tissues [37],[38]. However, nothing is known of the mechanism by which *A. tabida* eggs adhere to host tissues. Parasitism success of *C. sesamiae* in the host *Busseola fusca* relies on the suppression of host immune defenses by polydnaviruses (PDVs) injected with the egg. Differences in the sequence of one PDV gene (CrV1) and in its level of expression in the host exist between virulent and avirulent parasitoids [34],[39] but CrV1 role in virulence of *C. sesamiae* has not been demonstrated.

In *L. boulardi*, two virulence factors have been characterized and extensively studied in the venom, one in each of two lines, ISm and ISy, that display opposite virulence properties towards *Drosophila* host species [15],[16],[23],[31],[40]. *L. boulardi* then certainly provides the best parasitoid model to address the issue of the molecular bases of variation in virulence of an immune suppressive parasite.

The LbGAP protein appears as a major band in venom protein electrophoretic patterns of all strains virulent against *D. melanogaster* analyzed to date, but it was not observed in the avirulent ISy line [23]. The demonstration that LbGAP represents a major toxin, sufficient for parasitoid virulence toward this host species, comes from experimental evidence that the proteins eluted from this band, when injected into host larvae, conferred the same protection to avirulent parasitoid eggs as injection of total ISm venom [23],[24],[29]. Here, we show that this protection is abolished if the venom is previously incubated with an anti-LbGAP antibody. Also, injection of the antibody into host larvae before parasitism significantly decreases the success of ISm parasitoids. LbGAP is thus the main factor responsible for protection of *L. boulardi* eggs in resistant *D. melanogaster* hosts, and it is necessary for parasitoid virulence. This might explain why virulence is reported to be controlled by a single chromosomal factor despite the presence of several proteins in the venom [41]. Variation in LbGAP is then likely responsible for most of the variation of virulence between *L. boulardi* strains.

Here, we show that a gene homologous to *LbGAP* (*LbGAPy*) is expressed in venom-producing tissues of the avirulent parasitoid line and that the protein is present in the venom. The previous extensive characterization of LbGAP [15],[23],[24] thus provided a unique opportunity to assess whether variation of virulence is due to quantitative differences in this toxin or to qualitative changes that would impair binding to its targets or reduce its activity.

LbGAP and LbGAPy deduced amino acid sequences are 89% identical, and both contain a signal peptide and a conserved GAP domain. The recombinant proteins have a similar level of GAP activity and they interact with the same host targets, in agreement with the conservation of critical interacting amino acid residues [15]. Altogether, no qualitative difference was observed between LbGAP and LbGAPy toxins in our functional assays that could explain variation in virulence between virulent and avirulent parasitoids. Occurrence of *in vivo* differences in protein binding or activity in fly hemocytes cannot be ruled out but is very unlikely.

We previously showed that LbGAP is present in high amounts inside lamellocytes of ISm parasitized hosts [15]. Following parasitism by ISy, LbGAPy could also be detected inside host lamellocytes, but in a much lower number of cells and, when present, in a much lower quantity compared to LbGAP. Moreover, the morphology of LbGAPy-containing lamellocytes remained unchanged, in agreement with the previous observation that LbGAP quantity in a cell correlates with the degree of shape alteration [15]. LbGAP and LbGAPy are then both able to “enter” host hemocytes but the quantity of LbGAP in ISm venom is 60-fold higher than that of LbGAPy in ISy venom. This difference is sufficient to explain the difference in the amount of the two toxins inside host lamellocytes. A different rate of entry between LbGAP and LbGAPy in host cells cannot be ruled out but it would not be detected given the low quantity of LbGAPy in ISy venom. Such a high difference in quantity is probably responsible for the absence of detection of LbGAPy in ISy venom in a previous study [23]. Interestingly, the toxin amount in venom was twice lower in F1 hybrids than in ISm parasitoids while F1 hybrids were as virulent as ISm on *D. melanogaster* resistant flies. This also supports the idea that a minimal quantity of LbGAP is necessary for *L. boulardi* success in resistant *D. melanogaster*, and suggests the possible existence of a threshold effect on LbGAP/LbGAPy quantity in the virulence phenotype.

The production of high amounts of LbGAP is probably under strong selection in Mediterranean areas where resistant *D. melanogaster* are often encountered as hosts [13]. The selection in tropical Africa would be relaxed due to the occurrence of alternative host species and to a possible cost of LbGAP overproduction. The reason why no resistance to virulent parasitoids has been described yet in *D. melanogaster*, while resistance to avirulent parasitoids is found at high frequencies, might be that resistance to injection of high amounts of LbGAP is difficult to evolve. Rac GTPases, the targets of LbGAP, are highly conserved proteins due to their key role in cell functions and target modification is unlikely to evolve. Some removal of LbGAP from the host hemolymph is performed via phagocytosis by host plasmatocytes [15]. However, the high quantity of the LbGAP injected, together with the fact that the toxin quickly enters host lamellocytes, may encompass phagocytosis capacities. Evolution of resistance would thus require evolution in the potential of detoxification by host hemocyte cells or of degradation of the toxin, for instance via host proteases. A connected question is the reason why LbGAP is not efficient on *D. yakuba* lamellocytes in spite of the total conservation of its Rac targets between the two host species. Answers might involve differences in their intrinsic potential of degradation of foreign proteins, the higher number of hemocytes cells recorded in *D. yakuba* [42], or differences in lamellocytes that would influence the capacity of “entry” of LbGAP.

Differences in the RacGAP protein amounts in venom of the two parasitoid lines were correlated with differences in the amount of LbGAP/LbGAPy mRNA in venom-producing tissues, while we found no difference in gene copy number using genomic DNA from venom tissues or residual bodies. This allowed us to conclude that variation in the RacGAP toxin between virulent and avirulent strains is mainly quantitative. It likely results from differences in regulation of gene transcription in venom-producing tissues, even if the hypothesis of a difference in *LbGAP* and *LbGAPy* mRNA stability cannot be totally ruled out.

One of the characteristics of most parasitoid venom proteins is their high amount in venom compared to other tissues, which often correlates with a high level of expression of their coding genes [16],[43]–[50]. Here, we found a much higher mRNA level of the RacGAP toxin in venom-producing tissues of the two parasitoid lines than in the rest of the body. Such a tissue-specific change of expression is one of the traits likely selected in the process of re-use of a protein as a virulence factor, because such factors need to be delivered into the host via injection of venom at each oviposition event [51]. Transcription of the LbGAP/LbGAPy gene is thus regulated both in a tissue-specific manner and differently between virulent and avirulent strains.

Changes in gene regulation are now believed to play a prominent role in evolution of biological diversity. Regulatory factors that control gene expression are mainly transcription factors that bind to *cis*-regulatory elements in the upstream sequences of the gene, and microRNAs (miRNA) [52]. Specific gene expression in parasitoid venom-producing tissues might be driven by the availability of transcription factors that are tissue-specific [53]. Differences in the binding level of transcription factors to *cis*-regulating sequences might explain the different level of expression between parasitoid lines. They could originate either from evolutionary changes in these sequences, as recently reported at the inter-specific level [54] or from variation in accessibility of *cis*-regulating sequences [55]. In *L. boulardi* F1 individuals between ISm and ISy strains, it is the *LbGAP* allele that is overexpressed in the venom, the transcription of the *LbGAPy* allele remaining very low. This result supports the hypothesis of variation in *cis*-regulation of LbGAP expression. Full characterization of the mechanisms involved in regulation of LbGAP transcription will involve cloning and comparing upstream gene sequences and promoter regions between parasitoid strains, characterizing binding of transcription factors, and if necessary analyzing the miRNA expressed in venom protein-secreting cells since changes in miRNA-mediated gene regulation [52] can allow a quick and reversible diversification of the gene expression program. This would provide insights in understanding the mechanisms of transcriptional evolution, currently under active investigation, at the intra-species level.

The regulation of transcription of a venom factor reported here is the first described mechanism at the origin of intraspecific variation in immune suppressive properties of a parasite. An open area of research is now to define how common is this mechanism and whether its occurrence is linked to the nature of the virulence factors, is in relation with the taxonomy, or might be parasitoid specific. Parasitoids are major auxiliaries in the control of insect pests and their host range and specificity are widely discussed in the literature [56],[57]. Estimations of the potential for evolution of virulence molecules and acquisition of new virulence factors in a parasitoid species are essential information to understand and improve the results of biological control assays.

## Materials and Methods

### Biological material

The origins of the *L. boulardi* ISy (Gif stock number 486) and ISm (Gif stock number 431) isofemale lines have been previously described [41]. Briefly, ISy derives from a single female originating from Brazzaville (Congo) while ISm derives from a single female collected in Nasrallah (Tunisia). ISm females are highly virulent against *D. melanogaster* while parasitism success of ISy females depends on the resistant/susceptible genotype of the host [12]. *L. boulardi* F1 hybrid females were obtained from crosses between ISm females and ISy males. Both ISm and ISy lines, as well as F1 hybrids, were reared on a susceptible *D. melanogaster* strain (Gif stock, number 1333), at 25°C. After emergence, adults of both lines were kept at 18°C on agar medium with honey.

### Parasitism experiments

For parasitism experiments, the *D. melanogaster* YR strain (Gif stock, number 1088), resistant to *L. boulardi* ISy parasitoids, was used as host [32]. In each experiment, 30 second-instar host larvae (L2) were parasitized during 4 hours by one parasitoid female. The encapsulation ability was estimated 48 hours later by counting the number of encapsulated eggs after dissection of late third-instar larvae. Virulence was expressed as the ratio of non-encapsulated parasitoid eggs to the number of mono-parasitized hosts.

### Injection experiments

The first injection experiment was performed using freshly collected venom from ISm parasitoid females. The venom apparatus of 20 individuals were carefully removed and placed in 20 µl of Ringer's saline solution. The sample was homogenized manually in an Eppendorf tube and the extract was centrifuged at 500×g, 4°C for 5 min to eliminate the cellular debris. 10 µl of the supernatant were then incubated during one hour at 4°C either with the preimmune serum or the specific anti-LbGAP polyclonal antibody, both diluted 1∶10. Finally, 20 nl of the incubated venom were injected in L2 *D. melanogaster* YR larvae using a Nanoject II injector (Drummond Scientific Company, Broomall, PA). Approximately 180 larvae were injected then parasitized by ISy parasitoid females as described above but during a two hours period.

In the second injection experiment, 20 nl of the preimmune serum or of the specific anti-LbGAP polyclonal antibody, both diluted 1∶10, were injected in approximately 180 L2 *D. melanogaster* YR larvae which were then parasitized by ISm females as described above.

### Cloning and sequence analysis

To obtain the sequence of the *LbGAP*y cDNA, total RNA was extracted from *L. boulardi* ISy venom producing tissues using the TRIzol reagent (Invitrogen). RT-PCR was then performed using two specific primers designed from the cDNA sequence of *LbGAP* [23], 5′-CATAATTTTCAAATCTTCAACTTTTTTAGA-3′ and 5′-TTAGTCTCTGCACTTTTTCTCA TTTGATGT-3′. The amplified fragments were cloned into the pCR2.1-TOPO vector (Invitrogen) and sequenced.

Pairwise sequence comparisons were performed using the EMBOSS program Needle at EMBL-EBI (http://www.ebi.ac.uk/emboss/align/). The search for domains was performed using CDD (Conserved Domain Database) at NCBI (http://www.ncbi.nlm.nih.gov/Structure/cdd/cdd.shtml) and InterProScan at EMBL-EBI (http://www.ebi.ac.uk/Tools/InterProScan/). Occurrence and position of the signal peptide cleavage site were predicted using SignalP at CBS (http://www.cbs.dtu.dk/services/SignalP/) and Phobius at SBC (http://phobius.sbc.su.se/).

### *In vitro* GAP assays

*In vitro* GAP assays with recombinant GST-LbGAP and GST-LbGAPy proteins were performed in triplicates using the RhoGAP Assay Biochem Kit from Cytoskeleton Inc. P values were generated by ANOVA followed by pairwise comparisons using pairwise t tests performed with the R software package (http://www.r-project.org/).

### Yeast two-hybrid analysis

The *LbGAP*y cDNA was inserted into the pGADT7 vector by homologous recombination in yeast strain JD53 (MATa, his3–200, leu2–3112, lys2–801, trp1–63, ura3–52). Interactions between LbGAP and mutated forms of RhoA, Rac1, Rac2 and Cdc42 GTPases were then examined individually by mating as previously described [15]. The plasmids expressing GTPase proteins were tested against the pGADT7 empty vector and the pGADT7-T control vector that encodes a fusion between the GAL4 activation domain and SV40 large T-antigen. Reciprocally, the plasmid producing LbGAPy was tested against the pLex-Lamin control vector. Interactions between LbGAP and Rac1 and Rac2 GTPases were used as positive controls [15]. Interactions were first tested by spotting five-fold serial dilutions of cells on minimal medium lacking histidine and supplemented with 3-amino-triazole at 0.5 mM to reduce the number of false positives. Quantification of ß-galactosidase activity in liquid assays was then performed according to the Yeast Protocols Handbook PT3024-1 (Clontech Laboratories, Inc.) except that yeast cells were lysed using glass beads (Sigma). P values were generated by ANOVA followed by pairwise comparisons using pairwise t tests performed with the R software package.

### Production of recombinant proteins

GST-LbGAP was produced using a previously obtained construct [23] corresponding to the full-length LbGAP cDNA (without the signal peptide) cloned into the pGEX-5X-1 vector (GE Healthcare). For production of GST-LbGAPy, a cDNA fragment corresponding to the mature LbGAPy protein was amplified by RT-PCR from total RNA from ISy venom-producing tissues. The amplified fragment was cloned into the pGEX-4T-2 vector (GE Healthcare) using *Eco*RI and *Xho*I restriction sites. Competent BL21 *Escherichia coli* cells were subsequently transformed with the recombinant plasmids. The production and purification of the GST-LbGAP and GST-LbGAPy fusion proteins and GST alone were performed according to the GST Gene Fusion System Handbook (GE Healthcare).

### Immunocytochemistry

Immunocytochemical experiments were performed as previously described [15], using a rabbit anti-LbGAP polyclonal antibody [23] and Phalloidin-X5-FluoProbe 505 (Interchim) to visualize F-actin.

### Quantitative real-time RT-PCR

Total RNA was isolated either from dissected venom apparatus or from the rest of the female bodies (without venom-producing tissues) using the TRIzol reagent (Invitrogen), and reverse-transcribed using the iScript cDNA Synthesis Kit (BioRad). qPCR reactions were then carried out on an Opticon monitor 2 (BioRad) using the Absolute qPCR SYBR MasterMix Plus for SYBR Green I No ROX (Eurogentec) and the specific primers 5′-TGAAAGGGCGAATAATTGATG-3′ and 5′-TTTGGTGGAAGTTTGGAA-3′ for *LbGAP* and *LbGAP*y, respectively. PCR conditions were as follows: 50°C for 2 min, 95°C for 10 min, followed by 40 cycles of 95°C for 30 s, 60°C for 30 s and 68°C for 30 s. Each reaction was performed in triplicate and the mean of three independent biological replicates (venom-producing tissues) or two independent biological replicates (rest of the bodies) was calculated. All data were normalized using the *ITS2* (Internal Transcribed Spacer 2) ribosomal sequence as a control and results were analyzed using the ΔCt method. P values were generated by Student's t test with the R software package.

### Quantitative real-time PCR

Genomic DNA was isolated either from dissected venom apparatus or from the rest of female bodies (without venom apparatus) using the DNeasy Blood & Tissue Kit (QIAGEN). qPCR reactions were then carried out as described above. Each reaction was performed in triplicate and the mean of three independent biological replicates was calculated. All data were normalized using the *ITS2* ribosomal sequence as a control and analyzed using qBase software (http://medgen.ugent.be/qbase/). P values were generated by ANOVA followed by pairwise comparisons using pairwise t tests performed with the R software package.

### PCR and RT-PCR experiments with primers specific to *LbGAP* and *LbGAPy*

Genomic DNA isolation, total RNA isolation, and reverse transcription were performed as described above. The specific primer pairs were 5′-CTCCTGAAGACAGTGTAGAAATTATTC-3′ and 5′-GAATTTTTGAAACATCACTCGAAATA-3′ for *LbGAP* and 5′-GCTCCT AAAGACAGTATAGCAATTGTTA-3′ and 5′-AGATTAATTGAAACATCATCCGAAAT-3′ for *LbGAPy*. PCR was performed using GoTaq DNA Polymerase (Promega) as follows: 94°C for 2 min, followed by 35 cycles of 94°C for 30 s, 60°C for 30 s and 72°C for 45 s. Serial 1∶10 dilutions were used for cDNA templates. Amplification products were analyzed on a 2% ethidium bromide-stained agarose gel.

### Blotting experiments

For dot blot experiments with recombinant GST-LbGAP and GST-LbGAPy proteins, serial 1∶5 dilutions, starting from 10 ng of recombinant protein, were blotted onto a nitrocellulose membrane. GST alone was used as a negative control. Non-specific binding sites were blocked by overnight incubation at 4°C with TBST-2% milk buffer (20 mM Tris-HCl pH 7.5, 150 mM NaCl, 0.3% Tween 20). The membrane was probed 1 h with the LbGAP antiserum used at a 1∶5000 dilution. After three washes with TBST buffer, specifically bound antibodies were detected using anti-rabbit IgG horseradish peroxidase conjugate (Sigma) used at a 1∶15000 dilution for a 1 h incubation period. The membrane was revealed using the chemiluminescent Immobilon Western substrate (Millipore). Relative spot intensities were digitalized and quantified using GeneSnap and GeneTools softwares (Syngene).

For slot blot experiments comparing ISm and ISy extracts, 20 female venom apparatus were dissected in 20 µl of Ringer's solution and centrifuged for 2 min at 500×g. The supernatant was then diluted in 200 µl of denaturation solution (10 mM Tris-HCl pH 8, 100 mM NaPO4, 8 M Urea) and blotted onto a nitrocellulose membrane. For slot blot experiments comparing ISm and F1 hybrid extracts, only 5 female venom apparatus were used for each strain. The quantity of protein in the samples was determined using the Coo Protein Assay (Biorad) and found to be equivalent between the different parasitoid strains.

### Accession numbers

The GenBank accession number for the nucleotide sequence of LbGAPy is GU300066.
